# Crop fertilization affects pollination service provision – Common bean as a case study

**DOI:** 10.1371/journal.pone.0204460

**Published:** 2018-11-02

**Authors:** Davi de L. Ramos, Mercedes M. C. Bustamante, Felipe D. da Silva e Silva, Luísa G. Carvalheiro

**Affiliations:** 1 Departamento de Ecologia, Universidade de Brasília (UnB)—Campus Universitário Darcy Ribeiro, Brasília, D.F., Brazil; 2 Centro de Desenvolvimento Sustentável, Universidade de Brasília (UnB)–Campus Universitário Darcy Ribeiro, Brasília, D.F., Brazil; 3 Instituto Federal de Mato Grosso (IFMT)—Av. Sen. Filinto Müller, Cuiabá, MT, Brazil; 4 Departamento de Ecologia, Universidade Federal de Goiás, Goiânia, Brazil; University of New England, AUSTRALIA

## Abstract

The demand for insect-pollinated crops is increasing. Conventional agricultural intensification heavily relies on increased input of fertilizers, which can have negative effects on local biodiversity. Such effects may be particularly accentuated in biodiversity hotspots that are naturally nutrient-poor. Ecological intensification of farming, i.e. practices that increase production through the increase of ecosystem services, emerges as an alternative to conventional intensification. For example, practices that boost abundance and diversity of crop pollinators can lead to substantial increases in cropland productivity. However, little is known about the synergisms and trade-offs between fertilizer input and such ecological intensification practices. Here we investigate interactive effects between fertilization practices and the provision of ecosystem services in a biodiversity hotspot where conventional agriculture is rapidly expanding (Brazilian savannas). We focus on a highly nitrogen-demanding crop species that benefits from pollinators (the common bean, *Phaseolus vulgaris* L.), for which nitrogen input greatly varies in the study region. Our findings show that positive effects of native pollinators on crop yield are most accentuated under low inputs of nitrogen (e.g. equal to or below 72kg ha-1). This interactive effect could be due to changes in flower visitor community composition or behaviour. Our study also suggests that landscape management practices that minimize isolation from patches of natural vegetation and maximize its cover nearby (within 500 meters) of production areas can increase pollinator and biocontrol agent abundance and richness. Overall, these results suggest that ecological intensification is a valuable alternative for common bean production in Brazil, and potentially other regions of the world. Land productivity can be enhanced if an adequate balance of chemical inputs and landscape management is achieved.

## Introduction

Increasing agricultural farmland productivity while minimizing environmental damage is essential to reach sustainable development. On one hand, the increasing human population and growing demand for healthier diets has stimulated fruit and vegetable production, especially in regions of the world where food insecurity is greatest [1, 2]. On the other hand, there is urgency to reduce environmental degradation and stop biodiversity loss [3]. Promoting ecological intensification, whereby the productivity of agricultural land is increased through the enhancement of ecosystem services [4], rather than by conventional agriculture intensification (e.g. agrochemical use, cropland expansion), that are key drivers of environmental degradation (see [5, 6]), is crucial.

Biotic crop pollination is an important ecosystem service [7, 8, 9], influencing both local and global markets (e.g. [10, 11]). Farming practices aiming to increase the abundance and diversity of crop pollinators (e.g. maintenance of nesting habitats and floral resources within farmland, restricted use of pesticides) can benefit yield and quality of crops [9]. These practices may also benefit other ecosystem services providers important for agriculture, such as natural enemies that act as biocontrol agents [12]. Yet, few studies have evaluated synergisms and trade-offs between conventional (e.g. fertilizer input) and ecological intensification practices.

Farming practices involving chemical fertilizer application (e.g. phosphorus, P, and nitrogen, N) are used worldwide, and benefit crop productivity [13]. However, in the hope to overcome production deficits, farmers commonly apply fertilizer doses far above than recommended dosage [13] (farmers personal communication). The presence of these nutrients in excess, particularly nitrogen, can affect several reproductive traits of plants (e.g. quantity and quality of flower resources, [14, 15]). Consequently, the physiology, behaviour, abundance and diversity of flower visitors can be altered [15, 16]. Previous studies suggest that benefits from pollinators are accentuated at lower N levels (e.g. in oilseed rape, [17]), but it is unclear how generalized such effects are. Understanding interactive effects between fertilization practices and ecosystem services is essential to develop policies and management strategies that harness the power of ecological processes and functions to benefit both biodiversity and crop production.

This work aims to understand how management practices within farmland (nitrogen application and maintenance of fragments of natural habitat) affect ecosystem service provision. While the study focuses on flower visitors, among these, pests and two different types of ecosystem service providers were detected: species that act mainly as pollinators (hereafter ‘pollinators’), and species that act as predators and are recognized biocontrol agents of crop pests (hereafter ‘biocontrol agents’). We use common bean (*Phaseolus vulgaris* L.) as a study system, a crop of great importance for food security in Brazil and in many other regions of the world [18, 19]. As this species is known to benefit from pollinators [20, 21], we expect that increases in density and richness of pollinators and biocontrol agents enhance bean production, regardless of the origin of the species (i.e. native or exotic, here considered *Apis mellifera*) (hypothesis 1). In addition, as shown by Marini et al [17], we expect that pollinators’ benefits to crop yields will increase as N fertilizer input reduces (hypothesis 2). Finally, as the supply of soil nutrients can affect the flowering, floral resources, and consequently their attractiveness to floral visitors [15, 16], we expect that the composition of ecosystem service provider communities (i.e. pollinators and biocontrol agents) will change with increasing N input, particularly near patches of natural vegetation (hypothesis 3).

## Materials and methods

This study was conducted in private property and we confirm that the owners of the land gave permission to conduct the study on this site. No specific permissions were required for the plant (crop) and insect collections, and the field studies did not involve endangered or protected species. The study area is embedded in the “Cerrado” biome (savannah), which is a biodiversity hotspot [22] greatly affected by agrobusiness expansion [23], and where common bean (*Phaseolus vulgaris* L.) is commonly grown (Table A and Figure A in S1 File). Soils in the Cerrado are naturally poor in nutrients such as phosphorus and calcium [24]. The climate is tropical (Köppen Aw), characterized by dry (May to September) and wet (October to April) seasons.

Brazil is the largest producer and consumer of common bean in the world [25]. Farmers artificially inoculate *Rhizobium* (personal communication), which reduces the need of inorganic fertilizer [26] and apply inorganic and organic fertilizers which are frequently above the recommended dosage (see Table A in S1 File). Although common bean can self-fertilize to some extent [27], pollinators enhance yield and seed quality of this species (e.g. [20, 21]). This crop is affected by several pests, (e.g. *Bemisia tabaci*, Hemiptera) [28] which can serve as larval resources to flower visitor species (e.g. hoverflies).

Field surveys (November–January in 2015/2016 and 2016/2017) were focused on the cultivar ‘BRS Estilo’ (commercial group ‘carioca’), which is widely used by farmers in the studied region. Thirty-five sampling sites (27 in 2015/2016 and eight in 2016/2017, each 50x50m) were selected throughout 11 extensive monoculture fields (between 36 and 236 hectares) belonging to nine farmers. The minimum distance between each field was 1km. As for pollinators species (including *Apis mellifera*) foraging activity is mostly done within a range of 1km from their nesting/oviposition areas [29, 30], the minimum distance of 1 km ensures different pollinator communities. We selected multiple sites within the fields (two to six sampling sites per field, depending on the size of the field) in order to cover a gradient of distance to natural habitat (18 to 1152 m) (see Table A in S1 File) and maintaining a minimum distance between sites of 300 meters, a distance at which accentuated changes in abundance and diversity of crop pollinators are expected (e.g. [31]). All sampling sites had similar plant density (ca.12 plants/m2) and soil texture, colour, and chemical properties (see Table B and C in S1 File). All fields used no-tillage, with intensive use of pesticides during plantation and during flowering period, and the main rotation crops were corn (*Zea mays*) and soybean (*Glycine max*). Details on soil fertilization input and cultivation systems were obtained directly from the farmers, who keep regular records of their activities (Table B in S1 File). Total input of applied fertilizers (N and P) was calculated for each field based on the concentration of each of the nutrients in the elemental form applied (see details in [32, 33]). As P input was relatively similar across the sites, only the N input (which varied from 36 to 130 kg / ha) was used in data analyses. Soil pH, water, organic matter, were similar across sites (see Table C in S1 File).

Using satellite images and a geographical information system (QGIS), two landscape metrics that are known to influence crop pollinators were calculated for each study site: distance to natural habitat and vegetation cover (%). The predominant vegetation around the fields is cerrado *sensu stricto*, characterized by predominance of herbaceous and shrub stratum. Margins of semi-natural areas with more than five meters width were considered in such calculations. Depending on their functional traits (e.g. foraging ability, sociality), pollinators may respond to changes at different spatial scales [34]. Therefore, we calculated vegetation cover in four circular areas around each sample site, with radii of 0.5, 1, 1.5 and 2 km (Table D in S1 File).

### Flower visitation data

Following the methodology proposed by Vaissière et al. [35] in each sample site, we first quantified the total number of flowers in anthesis and flower visitors along two parallel transects (25x1m, each flower within transect was observed for ca. 30 seconds). Data were collected during morning (09h00 to 12h30) and afternoon (12h30 to 16h00), maintaining a minimum time interval of three hours between surveys in a given site (so each site was sampled twice within a single day of the peak of flowering). Environmental conditions were measured in all surveys (temperature between 21 to 37°C, humidity between 32 and 88%, all surveys occurred when there was no precipitation). All insects that touched the reproductive organs of flowers were considered “legitimate flower visitors”. Robbing events (i.e. perforation of the corolla to extract nectar without having contact with the reproductive organs), were also recorded. Whenever an insect was observed, a description of the visitor (Order, size and colour pattern) was also recorded (morphospecies). Afterwards, insects were captured along the transect, and later identified by taxonomists.

While the sampling method was developed specifically to detect pollinators, many species that act as herbivores and seed predators during larval stages visit flowers in their adult stage were detected. Although all these floral visitors may have contributed to pollination to some extent, some have a different main function having negative (crop pests) or positive (biocontrol agents) additional effects. Consequently, in our specific study, flower visitors were divided into three main functional groups based on species biology: pollinators, species that may act as biocontrol agents and species that may act as crop pests (see Table E in S1 File). Pollinators included all bees as well as one syrphid fly species (*Palpada vinetorum* Fabricius, 1798) that does not act as predator during the larval stage [36]. In the group ‘potential biocontrol agents’, we included wasps (Hymenoptera: Vespidae and Pompilidae) which typically predate larvae of Lepidoptera and Coleoptera [37], and one Syrphidae morphospecies that belongs to a genus that feeds on agricultural pests that affect common bean (*Allograpta exotica* cf.). Pests included several Lepidoptera, Coleoptera and Hemiptera species which are known to attack common bean or other crop species [28]. Pollinators were further divided into exotic (i.e. honeybee, *Apis mellifera*) and native pollinators. Wild populations of honeybees occur in the study region and none of the farmers involved in this study managed honeybees. However, given the vast foraging range of this species, we were not able to identify the status of the populations (wild or managed) from which honeybees detected visiting common bean in our study region came from.

As the number of flowers observed varied among plots, and sampling effort is directly proportional to total number of flowers observed, we calculated (i) flower visitor density and (ii) species density, and used these variable in data analyses of productivity. To do that we divided the total number of flower visitors for each group and species by the total number of flowers observed. Only species that were not crop pests were considered in richness calculations. Richness estimations also considered any uncollected morphospecies, which description did not match with the collected species.

### Production data

Production data were collected ca. 90 days after the date of planting, during the fruiting period, on the exact same place where flower visitation surveys were done. On each sampling site, 15 crop plants were randomly selected. For each individual selected, all pods produced (including thin pods with no beans, due to lack of ovule fertilization, or with aborted beans) were collected and stored in paper bags. The number of mature beans (> 3mm, following national marketing definition for common bean [38], all other beans were considered aborted) were counted and placed in a 65°C oven until they reached humidity below 14% [39]. Afterwards, the beans were weighed. Overall productivity (yield) depends on number of flowers produced per area unit, and on the number of ovules fertilized per flower. Only the last is influenced by pollinators, and the first is highly dependent on soil fertilization [16]. As this work aimed to evaluate the effect of pollination services, we considered two production metrics: productivity per flower and overall land productivity (yield). As the first metric is independent of the total number of flowers, it better represents the effect of pollination. Number of flowers observed during flower surveys were done is not necessarily correlated with the total number of flowers produced over time in that plot. Therefore, we considered the number of pods (including those undeveloped) to be the best proxy of the number of flowers produced throughout the flowering season. Productivity per flower was, hence, calculated by dividing the total bean weight per plant by the total number of pods. Overall land productivity was calculated by multiplying the average bean weight per plant by the average number of plants per square meter in our study fields (see above) and converted to kg per ha. Overall production deficit was calculated as the ratio between the productivity per flower (of each sampling site) and the maximum value of productivity obtained in the study region, as calculated by Garibaldi et al. [9].

### Statistical analyses

Linear mixed-effects models (LMM) were used to evaluate the influence of the density of the different insect groups (i.e. native and exotic pollinators, biocontrol agents and pests) and overall richness (here, species density) on bean productivity. To account for the spatial and temporal aggregation of sampling points data, ‘field’ within ‘farmer’ and ‘year’ were included as random variables. We ran the analyses for the two productivity variables: productivity per flower and overall land productivity. Model assumptions were checked and to account for the typical sigmoidal behaviour of production (i.e. have established maximum and minimum values), and adapted logi transofrmation was applied to normalize residuals, using the maximum value of productivity detected in field (rounded up) as the top asymptote.

To test if higher richness and density of ecosystem service providers (pollinators and biocontrol agents) increased bean productivity and if the effect of density of pollinators was boosted by richness (hypothesis 1), density of native, exotic (i.e. *Apis mellifera*) pollinators, biocontrol agents and visitor richness were included as fixed terms. In addition, two-way interaction between density of pollinators and richness was also included as fixed terms. To test if pollinator benefits to crop yield increase as N fertilizer input reduces (hypothesis 2), interactions between pollinator density (native and exotic) and N input were also considered. To identify the terms that most contributed to explain bean productivity, a model selection procedure was applied, based on the Akaike Information Criteria corrected for small sample size (AICc) [40]. Whenever several models had equally good predictive power (i.e. several models with ΔAIC below 2), we applied model averaging. Only models with ΔAICc lower than 2 were considered in the calculation of the average estimates, and whenever a variable was not included in a model we assumed an estimate equal to zero.

To check if sites with extreme values of N input (with high and lower N input) were driving the results on the effects of N input on bean production, we ran additional models after removing extreme values (sampling points with 36 or 130 kg N/ha). This sensitivity test also allowed the detection of thresholds above which the effect of nitrogen is no longer observed in this region.

To test if the community composition (abundance of each group and overall richness) of ecosystem service providers and probability of pest occurrence changes with increasing N input, particularly near patches of natural vegetation (hypothesis 3), we used Generalized Linear Mixed Models (GLMM, negative binomial distribution), considering the same random variables mentioned above. First, for each group of ecosystem service providers, we selected the most suitable spatial scale by comparing the AICc values of models with each vegetation cover variable. We then ran another GLMM including the selected vegetation cover variable, distance to natural habitat, the total input of nitrogen and two-way interaction between N input and vegetation cover. To check if landscape management practices that potentially benefit ecosystem service providers also had an effect on crop pests, we repeated a similar procedure using pest densities as the response variable. As density of pests (that forage on flowers) was extremely low, we analyzed this data as presence-absence, using a logistic regression (binomial distribution). All model selection procedures focused on the variables of community composition and also included climatic data (temperature, humidity, and wind speed at the time of the survey) and observation time as covariates. All statistical analyses were performed using R version 3.3.1 using the R packages “lme4” and “MuMIn” [40,41].

## Results

Productivity per flower and overall land productivity varied greatly between plots with deficit (i.e. relative difference between productivity in a given point and the maximum value of productivity obtained in the study region) up to 85% and 92% respectively (Table A in S1 File). During this study, 283 flower visitors were recorded, belonging to 5 orders and 33 species or morphospecies (see Table E in S1 File). Of those, 30.8% were native pollinators, 32.5% were the exotic honeybee (*Apis mellifera*), 31% were potential biocontrol agents and 5.6% were pests. Among the native pollinators, visitors known to be effective pollinators of common bean crops (e.g. bumblebees and carpenter bees, see [21, 42]) were detected. Although exotic honeybees acted sometimes as legitimate pollinators, in most visitation events (75%) they acted as nectar robbers. One species of Syrphidae (*Allograpta exotica* cf.) represented 90.8% of the insects within the biocontrol agent group. Among pests, a well-known pest in bean fields as well as other crops (*Helicoverpa zea*, Lepidoptera) was the most abundant (25%).

### Effect of ecosystem service providers and N input on bean production

Flower visitors and N input influenced both metrics of bean productivity, these effects being more accentuated for productivity per flower (Table 1).

**Table 1:** Effect of ecosystem services providers (pollinators and biocontrol agents) and nitrogen application on productivity per flower (g) and overall land productivity (kg ha-1) of common bean (*P*. *vulgaris*) farms.

**Response variable (Y)**	**Explanatory terms**				**Weight**	**AICc**	**∆AICc**
**Productivity per flower**	DN	DE	N	DN*N			
Model1	-	-	X	-	0.269	63.9	0.00
Model2	-	X	X	-	0.134	65.3	1.39
Model3	X	-	-		0.113	65.7	1.74
Model4	X	-	X	X	0.111	65.7	1.78
**Average model: log(**Y/(2-Y))=-1.32+(638.5-6.8*N)*DN+0.016*N-45.1*DE							
**Overall land productivity**	DN	DE	N	DN*N			
Model 1	-	-	-	-	0.287	110.3	0.00
Model 2	X		-	-	0.135	111.8	1.51
Model 3	-	X	-	-	0.124	112.0	1.68
**Average model: log(**Y/(5300-Y))= -014 + 54.25*DN –86.2*DE							

Models were selected based on the Akaike information criterion corrected to small sample size (AICc), and all models with a variation of AICc (ΔAICc) lower than 2 units were considered in the average model, the contribution being proportional to the model weight. As productivity models typically follow a sigmoid relationship (i.e. have established maximum and minimum values) we applied a logit transformation, using the maximum value of productivity per flower rounded to units (2 g) and productivity per ha rounded to hundreds (5300 kg.ha^-1^) as the top asymptote.

DN = Density of native pollinators

DE = Density of exotic pollinators (*A*. *mellifera*)

N = Nitrogen input

X = terms that were included in the models (Gaussian distribution for productivity per flower, and log transformed for overall land productivity to normalize residuals)

* = two-way interaction between explanatory variables or multiplication in the equation

**Fig 1:**
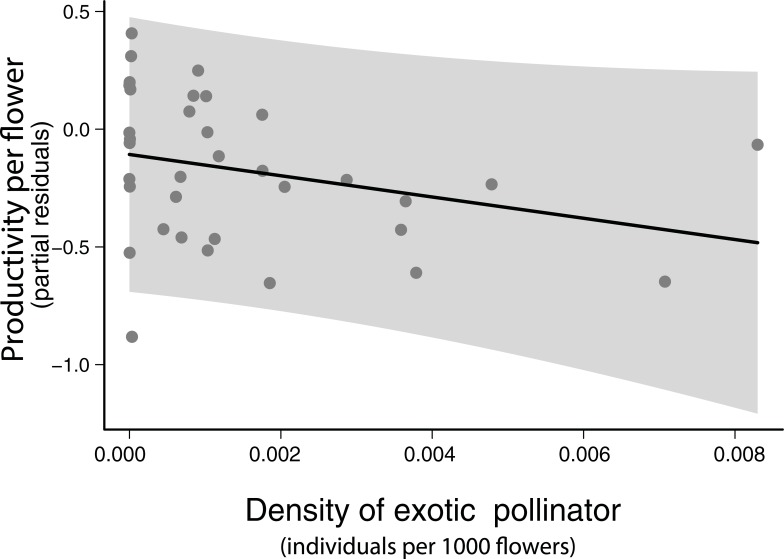
Effect of the density of exotic pollinators (i.e. *Apis mellifera*) on common bean productivity. Points represent partial residuals (i.e. variability not explained by the other variables included in the model). Model estimates are based on Model 2 from Table 1. Shaded area represents 95% confidence interval.

The positive effect of native pollinators on productivity was independent of flower visitor richness and no effect of biocontrol agents was detected, partly rejecting hypothesis 1. Contrary to our initial expectations, the density of exotic pollinators (i.e. *A*. *mellifera*) had a negative effect on productivity (Fig 1, Table 1).

**Fig 2:**
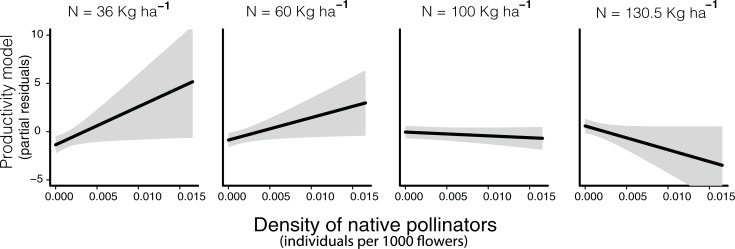
Estimated effect of density of native pollinators on common bean productivity under different levels of nitrogen (N) input. (N) Nitrogen application (varied from 36.0 and 130.5 kg ha-1 between fields). Model estimates are based on Model 4 from Table 1. Shaded area represents 95% confidence interval.

In agreement with hypothesis 2, pollinator benefit to productivity was greater when lower levels of N were applied, the effect being even negative at very high levels of N input (Fig 2). When using N input levels recommended for common bean plantations (in rotation with soya) in Cerrado (60 kg ha-1), and when no exotic pollinator is present, productivity per flower obtained in areas with high pollinator densities (0.0165 per flower) more than doubled (143% increase) in comparison with areas with no pollinators (0.82 vs 2.01 g per flower, see equations in Table 1). The effect of native pollinators on overall productivity was similar (i.e. 2464.8 kg.ha^-1^ with no native pollinators *vs* 5299.1 kg.ha^-1^, if pollinator density was kept high throughout the field) but no interactive effect with N application was detected. No positive effect of N was detected on overall productivity nor on number of flowers produced, instead a negative effect was detected fro flower productivity (Figure B in S1 File). Also, the positive effect of this nutrient on productivity per flower (Table 1) was no longer detected above 72 kg/ha (Table F in S1 File).

### Influence of fertilization practices and landscape characteristics on flower visitors

**Fig 3:**
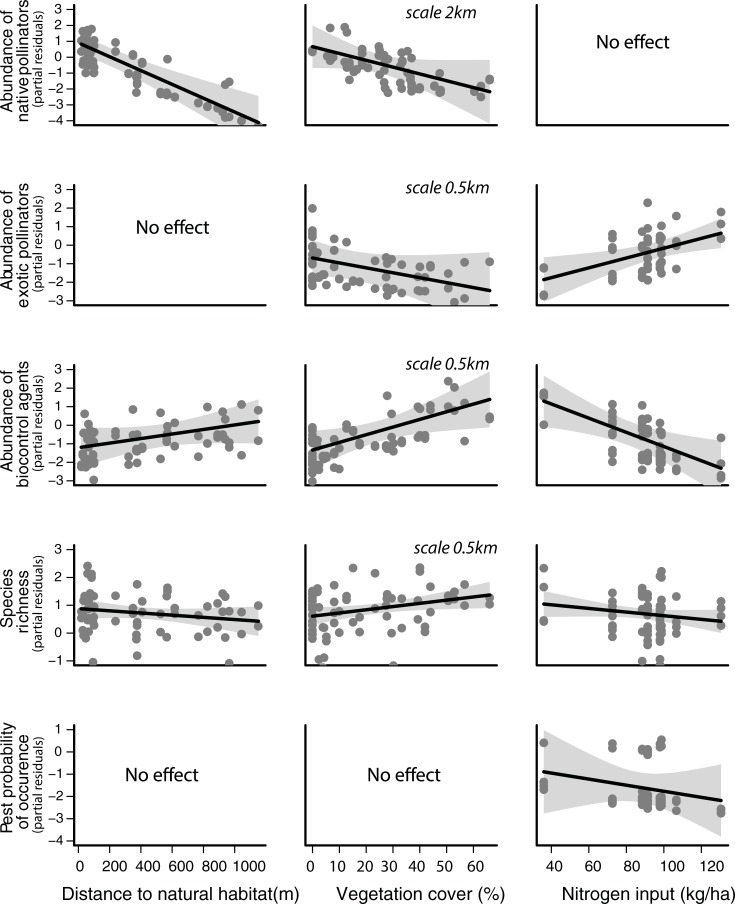
Effects of local management practices (nitrogen input and maintenance of native vegetation within agricultural landscape) on pollinators, biocontrol agents and species richness. The points represent partial residual. The graphs are based on the variables selected on the best models (ΔAICc < 2), having used always the best model that includes most environmental variables (see Table G in S1 File). For the analyses of abundance of native pollinators the vegetation cover scale used was 2000 meters. For the remaining variables, the vegetation cover scale used was 500 meters (see details of vegetation cover scale selection in Table D in S1 File). Shaded area represents 95% confidence interval.

Climate effects on flower visitors were diverse and were taken into account when assessing the effect of nitrogen and landscape (Table G in S1 File). Farms with higher nitrogen input had more visitation by the exotic *Apis mellifera* (which negatively affects production, see above), lower abundance of biocontrol agents, and lower richness of ecosystem providers and lower probability of pest occurrence (Fig 3). No overall effect on the abundance of native pollinators was detected. For information on species level responses to N input, see Figure C in S1 File. Number of flowers observed in surveys was positively correlated with the abundance of *A*. *mellifera*, biocontrol agents, pest occurrence, and overall richness.

As for landscape effects, the majority of the responsible variables (biocontrol agents, exotic pollinators, overall richness and pests) were most responsive to changes in vegetation cover at finer scales (500 meters, included in subsequent analyses), while native pollinators were most responsive at larger scales (2000 meters) (see Table D in S1 File). Native pollinator abundance was higher close to the margins with natural habitat, independently of the amount of natural habitat (Fig 3) available in the landscape (i.e. no interaction between cover and distance was detected). While landscapes with greater vegetation cover had fewer native pollinators, they also had more visitor species and higher abundance of biocontrol agents (Fig 3). When nitrogen input was low, the abundance of exotic honeybees declined with vegetation cover, and when N input was high the species abundance increased with vegetation cover.

## Discussion

Investment in ecological intensification, i.e. improving agricultural production via intensification of ecosystem service provision while minimizing the negative effects to biodiversity, is essential to achieve sustainable development. Our findings suggest that the density of ecosystem service providers (crop pollinators and other flower visitors that may also act as biocontrol agents) can be regulated not only by landscape but also by local (i.e. fertilization) management practices adopted by landowners. Below, we discuss the implications of our findings for agricultural production and biodiversity conservation.

### Effect of ecosystem service providers and N input on bean production

Despite farmers in our study region heavily investing on conventional intensification (chemical inputs and large extensions of monoculture), we detected important benefits of native pollinators on productivity (more than doubled, see Table 1). The strong variation in flower production, here detected will also affect productivity. Therefore, it is essential to maintain both flower production and pollinator density high to achieve optimal results in overall yield. The negative effect of the exotic honeybee on production is likely related to the fact that this species frequently acts as a nectar robber. Such behaviour may decrease flowers attractiveness to legitimate pollinators and even damage reproductive parts of the flower [43], consequently, having a negative effect on common bean production. However, the negative effect of honeybee abundance on native pollinator abundance here detected was not significant (see Fig D in S1 File). Overall, our results indicate that there is potential for improving crop yields through ecological intensification. While common bean was described by Klein et al. [7] as having little dependence on pollinators (i.e. 0–10%), that classification was based on information on a diverse group of species within the genus *Phaseolus*. At the time of Klein’s publication few studies on pollination efficiency of common bean existed, but those mentioning *Phaseolus vulgaris* highlight the potential importance of flower visitors for pollination [20, 44, 45], and one detected benefits from pollinators of 13–35% [20].

More recent studies working with different cultivars of common bean also detected important benefits in yield from pollinators in Cameroon [21] (production of fully developed beans more than double) and Kenya [42]. Thus, future studies should consider common bean as a crop with, at least, medium level of dependence on pollinators (i.e. 10–40%). The fact that, in our study, pollinator’s positive effects were most accentuated under lower N input and even became negative above 100 kg ha-1 (Fig 2) suggests an alteration of the plant’s investment strategy (reproductive versus vegetative development) under high N availability [46]. Indeed, in our study system nitrogen input had negative effect on overall flower production (see Figure B in S1 File). Increased N availability may have also changed the quality of resources (e.g. due to changes in essential amino acid, alkaloids, or flower shape) [14]. Such changes could alter flower’s attractiveness to the different flower visitor species affecting pollination effectiveness. For example, in our study, we detected a positive effect of nitrogen input on honeybee abundance (Fig 3) that as discussed above has a negative effect on common bean production. Further surveys and more detailed analyses would be required to evaluate changes in the behaviour of this species (legitimate visitor vs. robber) and on the visitation rates and behaviour of other species of visitors.

The interactive effect between pollinator density and N availability here detected corroborates with the findings of a previous study [17], which found a tendency for greater benefit from pollination under low N availability in canola. However, in their study, the extreme reduction in nitrogen availability used (150 vs. 0 kg ha-1) may have limited pollinator benefits. Fruit formation requires a high level of nutrients and many studies (including our own) show that below certain levels of N, production is constrained (see Fig 2). The positive effect of nitrogen input on productivity could be partially due to a positive effect on flower production (and consequently, on number of pods). However, the effect of this nutrient on flower production was slightly negative in our work (see Figure B in S1 File). The positive effect is hence more likely explained by the high requirement on nutrients for fruit production of our study species. This demand might be higher when cross pollination mediated by insects occurs, since beans produced might have greater nutritious content (see [47] for an example with almond species). However, the fact that no positive effect of N input was detected in the sensitivity tests run only with points with N input higher than 72 kg/ha suggest that farmers are using unnecessarily high levels of nitrogen that do not benefit fruit formation, and reduce the effectiveness of pollinators. Indeed, given that all but one of our study fields had planted soya (and on the field with no soya, another nitrogen fixing plant was planted, *Crotelaria* sp.) in the last 2 years (see Table B in S1 File), the recommended dosage for common bean plantations in Cerrado is ca. 60 kg ha-1 of nitrogen [33].

As for biocontrol agents, the most plausible reason for the lack of effect on bean productivity is the intensive use of pesticides. Such practices are probably regulating the abundance of pests, which was low in our field sites (2.8% of beans damaged). Further studies are needed to test whether this group of species would effectively maintain common bean crops pests at low abundance if pesticides were applied in lower levels. In other crop systems, pollinator richness improves the benefits of pollinator density (e.g. [48, 49]). The lack of positive effects of richness on productivity in our study system could be because such effect may be diluted in situations of low abundance of species [50], which limits the ability to detect synergistic positive effects between the two variables. Indeed, when comparing with *P*. *vulgaris* fields of small scale farmers in Africa [21, 42], the mean flower visitation rates detected in our study were very low (0.004 vs. ca 0.151 visits/flower). Although the flower visitors’ assemblages and sampling efforts of the two study regions likely differ, this striking difference is most likely due to the negative pressure resulting from the strong agricultural intensification in our study area (i.e. large monoculture areas with high chemical input of insecticides, including neonicotinoids, organophosphates, pyrethroids, acetates and carbonates, farmers’ personal communication).

### Influence of landscape characteristics and fertilization practices on flower visitors

The positive effect of vegetation cover within 500m radius on biocontrol agents and richness here detected suggests that small fragments of vegetation, adjacent to crop fields are essential to sustain the populations of these species. Such fragments are likely important nesting area for the bees and wasps detected in our study (see [51, 52]). For insects whose larvae can develop within crop fields (e.g. hoverflies), such patches can also be important alternative floral resources when common bean (and other crops) are absent from the landscape [53]. However, when common bean is flowering, those hoverflies depend less on native resources and more on availability of larval resources, possibly preferring to forage further away from natural habitat where they might be more protected from competition with other flower visitors and other pest natural enemies (e.g. wasps). This may explain the positive relationship between the density of biocontrol agents (a group dominated by one morphospecies of hoverfly, that is likely exotic, *Allograpta exotica* cf.) and distance to natural habitat (see also [54,55]. The positive effects of vegetation cover (2000km radius) on native pollinators could be due to a dilution effect, and reinforce the idea that conservation and restoration of natural habitats in the immediate surroundings of agricultural fields is important for ecological intensification of farming. The creation of hedgerows and flower strips may also bring important benefits facilitating movement of native pollinators [56, 57], and biocontrol agents (e.g. [58]).

Despite the variability of fertilizers application methods between farms, we detected a significant effect of nitrogen on visitor abundance and overall diversity of ecosystem providers. Nitrogen availability can alter amino acid content in pollen and nectar [14, 59], which can affect visitation of species that are attracted by flowers with specific amino acids contents (e.g. of proline, glycine, see [60, 61]). The fact that, in our study, different groups of visitors were affected differently by N availability (e.g. honeybee were positively affected, biocontrol agents negatively affected) suggests different preferences in terms of nectar or pollen quality. Indeed, Petanidou et al. [62] found that different species of flower visitors are differently affected by changes in amino acid content. Further studies evaluating the chemical composition of floral resources of common bean’ flowers would help to clarify their role as regulators of the effect of nitrogen on the attractiveness to the different bee species.

## Conclusions

Our findings suggest that ecological intensification is a promising pathway for one of the most important global crops, the common bean. Managing vegetation cover to increase the number of native pollinators in farms has the potential to increase production in this crop. However, this benefit is lost under high fertilizer application.

As benefits of nitrogen input not related with pollination also fade above certain threshold values (60 to 72 kg/ha in our study region), strategies that involve lower fertilizer dosage should be considered in addition to those already known to benefit crop pollinators (adequate use of pesticides, maintain flower diversity and nesting sites within farmland). These strategies have the potential to improve yield while reducing management costs and are particularly relevant in region, as the Brazilian savannas, which are very biodiversity rich and have naturally nutrient poor soils [63]. Current environmental laws for private properties in Brazil prioritize vegetation in proximity to water sources [64], a crucial ecosystem service for human wellbeing. In other countries, water and soil conservation, as well as mitigation of natural disasters are the main human-focused motivations considered in environmental laws [65]. Given the importance of agriculture for economy and food security pollinator conservation practices related with agricultural management should integrate future environmental policies. Further studies involving evaluations of quality of bean, and detailed economic evaluations of changes in management would be important to further motivate investment in ecological intensification practices.

## Supporting information

S1 File Combined supporting information file. **All supplementary tables (Tables A to F) and figures (Figures A to C) are listed.** Table A. Details of the study areas where flower visitation and yield data were collected. Table B. Management practices applied in each study area that can influence nitrogen (N) and phosphorous (P) content. Table C. Geographical location and soil characteristics of the study areas used in this study. Table D. Identification of the spatial scale at which the different flower visitor groups are most susceptible to changes of vegetation cover. Table E. Details of the species of insects found visiting common bean in our study region. Table F. Sensitivity analyses of the effect of ecosystem services providers (pollinators and biocontrol agents) and nitrogen application on productivity per flower and on overall land productivity. Figure A. Study area located in the central region of Brazil, showing the location of the 35 sampling sites used in this study. Figure B. Effect of the nitrogen input on overall flower production in common bean fields. Figure C. Distribution of pollinator species (native and exotic) and biocontrol agents along a gradient of increasing input of nitrogen (P1 had the highest N input and P35 the lowest).
(DOCX)

## References

[1] FAO, Food and Agriculture Organization. ‘Climate-smart’ agriculture, policies, practices and finances for food security, adaptation and mitigation. FAO, Rome; 2010.

[2] Garnett T, Appleby MC, Balmford A, Bateman IJ, Benton TG, Bloomer P, et al Sustainable intensification in agriculture: Premises and policies. Science. 2013; 341: 33–34. 10.1126/science.1234485 23828927

[3] CDB, Convention on Biological Diversity. Strategic Plan for Biodiversity 2011–2020. Available from: https://www.cbd.int/sp/targets/

[4] Bommarco R, Kleijn D, Potts SG. Ecological intensification: harnessing ecosystem services for food security. Trends in Ecology Evolution. 2013; 28: 230–238. 10.1016/j.tree.2012.10.012 23153724

[5] Kehoe L, Romero-Muñoz A, Polaina E, Estes L, Kreft H, Kuemmerle T. Biodiversity at risk under future cropland expansion and intensification. Nature Ecology & Evolution. 2017; 1: 1129–1135. 10.1038/s41559-017-0234-3 29046577

[6] Lanz B, Dietzd S, Swanson T. The expansion of modern agriculture and global biodiversity decline: an integrated assess. Ecological Economics. 2018; 144: 260–277. 10.1016/j.ecolecon.2017.07.018

[7] Klein AM, Vaissière BE, Cane JH, Steffan-Dewenter I, Cunningham SA, Kremen C, Tscharntke T. (2007). Importance of pollinators in changing landscapes for world crops. Proceedings of the Royal Society B. 2007; 274: 303–313. 10.1098/rspb.2006.3721 17164193 PMC1702377

[8] Garibaldi LA, Steffan-Dewenter I, Winfree R, Aizen MA, Bommarco R, Cunningham SA, et al Wild pollinators enhance fruit set of crops regardless of honey bee abundance. Science. 2013; 339: 1608–1611. 10.1126/science.1230200 23449997

[9] Garibaldi LA, Carvalheiro LG, Vaissière BE, Gemmill-Herren B, Hipólito J, Freitas BM, et al Mutually beneficial pollinator diversity and crop yield outcomes in small and large farms. Science. 2016; 351: 389–391. 10.1126/science.aac7287 26798016

[10] Gallai N, Salles J-M, Sattele J, Vaissière BE. Economic valuation of the vulnerability of world agriculture confronted with pollinator decline. Ecological Economics. 2009; 68: 810–821. 10.1016/j.ecolecon.2008.06.014

[11] Giannini TC, Cordeiro GD, Freitas BM, Saraiva AM, Imperatriz-Fonseca VL. The dependence of crops for pollinators and the economic value of pollination in Brazil. Journal of Economic Entomology. 2015; 108: 849–857. 10.1093/jee/tov093 26470203

[12] Steward PR, Shackelford G, Carvalheiro LG, Benton TG, Garibaldi LA, Sait SM. Pollination and biological control research: are we neglecting two billion smallholders. Agriculture & Food Security. 2014; 3: 1–13. 10.1186/2048-7010-3-5

[13] Mosier AR, Syers JK, Freney JR. Agriculture and the nitrogen cycle Assessing the impacts of fertilizer use on food production and the environment. Island Press, USA: Scope–The Scientific Committee on Problems of the Environment; 2004.

[14] Hoover SER, Ladley JJ, Shchepetkina AA, Tisch M, Gieseg SP, Tylianakis JM. Warming, CO2, and nitrogen deposition interactively affect a plant-pollinator mutualism. Ecology Letters. 2014; 12: 227–234. 10.1111/j.1461-0248.2011.01729.x 22221802

[15] Ceulemans T, Hulsmans E, Ende WV, Honnay O. Nutrient enrichment is associated with altered nectar and pollen chemical composition in Succisa pratensis Moench and increased larval mortality of its pollinator Bombus terrestris L. Plos one. 2017; 12: 1–15. 10.1371/journal.pone.0175160 PMC5390989 28406910

[16] Muñoz AA, Celedon-Neghme C, Cavieres LA. Arroyo MKT. Bottom-up effects of nutrient availability on flower production, pollinator visitation, and seed output in high-Andean shrub. Oecologia. 2005; 43: 126–135. 10.1007/s00442-004-1780-3 15583940

[17] Marini L, Tamburini G, Petrucco-Toffolo E, Lindström SAM, Zanetti F, Mosca G, et al Crop management modifies the benefits of insect pollination in oilseed rape. Agriculture, Ecosystems and Environment. 2015; 207: 61–66. 10.1016/j.agee.2015.03.027

[18] FAO, Food and Agriculture Organization. 2010. In: FAO web site. Available from: http://www.fao.org/faostat/en/#data/QC

[19] Silva HT, Costa AO. Caracterização botânica de espécies silvestres do gênero Phaseolus L. (Leguminosae). Embrapa Arroz e Feijão, 156; 2003.

[20] Ibarra-Perez FJ, Barnhart D, Ehdaie B, Knio KM, Waines JG. Effects of insect tripping on seed yield of common beans. Crop Sci. 1999; 39: 428–433. 10.2135/cropsci1999.0011183X0039000200022x

[21] Kingha BMT, Fohouo F-NT, Ngakou A, Brückner D. Foraging and pollination activities of Xylocopa olivacea (Hymenoptera, Apidae) on Phaseolus vulgaris (Fabaceae) flowers at Dang (Ngaoundere-Cameroon). Journal of Agricultural Extension and Rural Development. 2012; 4: 330–339. 10.1111/j.17485967.2011.00334.x

[22] Myers N, Mittermeier RA, Mittermeier CG, da Fonseca GAB, Kent J. Biodiversity hotspots for conservation priorities. Nature. 2001; 403: 853–858. 10.1038/35002501 10706275

[23] Strassburg BBN, Brooks T, Feltran-Barbieri R, Iribarrem A, Crouzeilles R, Loyola R, et al Moment of Truth for the Cerrado hotspot. Nature Ecology and Evolution. 2017; 1: 1–3. 10.1038/s41559-016-0001 28812670

[24] Oliveira PS, Marquis RJ. The cerrados of Brazil: ecology and natural history of a neotropical savanna. New York: Columbia University Press, pp. 398; 2002.

[25] Ferreira CM, Del Peloso MJ, de Faria LC. Feijão na economia nacional. Documentos. Embrapa Arroz e Feijão, 135 pp. 47; 2002.

[26] Dadson RB, Acquaah G. *Rhizobium japonicum*, nitrogen and phosphorus effects on nodulation, symbiotic nitrogen fixation and yield of soybean (*Glycine max* (LMerrill) in the southern savanna of Ghana. Field Crops Research. 1984; 9: 101–108. 10.1016/0378-4290(84)90016-9

[27] OECD, Organisation for Economic Co-operation and Development. Consensus document on the biology of common bean (Phaseolus vulgaris L.). OECD Environment, Health and Safety Publications. Series of Harmonisation of Regulatory Oversight in Biotechnology No. 59, Paris, France, JT03388473. 2015. Available at: http://isa.ciat.cgiar.org/genebank/urgweb_folder/files/unitfiles/OECD-SeriesonHarmonisationofRegulatoryOversightinBiotechnology-59.pdf

[28] Quintela ED. Manual de identificação de insetos e outros invertebrados pragas do feijoeiro Brasília: Embrapa Arroz e Feijão, 142, pp. 51; 2009.

[29] Gathmann A, Tscharntke T. Foraging ranges of solitary bees. Journal of Animal Ecology. 2002; 71: 757–764. 10.1046/j.1365-2656.2002.00641.x

[30] Greenleaf SS, Williams NM, Winfree R, Kremen C. Bee foraging ranges and their relationship to body size. Oecologia. 2007; 153: 589–596. 10.1007/s00442-007-0752-9 17483965

[31] Carvalheiro LG, Seymour CL, Veldtman R, Nicolson SW. Pollination services decline with distance from natural habitat even in biodiversity-rich areas. Journal of Applied Ecology. 2010; 47: 810–820. 10.1111/j.1365-2664.2010.01829.x

[32] Leikam DF, Lamond RE. Estimating manure nutrient availability Kansas State University 2003; 2562: 1–8.

[33] Sousa DMG, Lobato E. Cerrado: correção do solo e adubação. 2nd ed. Brasília: Embrapa Informação Tecnológica, pp.416; 2004.

[34] Steffan-Dewenter I, Münzenberg U, Bürger C, Thies C, Tscharntke T. Scale-dependent effects of landscape context on three pollinator guilds. Ecology. 2002; 83: 1421–1432. 10.1890/0012-9658(2002)083[1421:SDEOLC]2.0.CO;2

[35] Vaissière BE, Freitas BM, Gemmill-Herren B. Protocol to detect and assess pollination deficits in crops: a handbook for its use Global Action on Pollination Services for Sustainable Agriculture. Rome, Italy: Food and Agriculture Organization of the United Nations (FAO); 2011.

[36] Morales MN. Taxonomia das espécies do grupo Scutellaris do gênero Palpada Macquart (Diptera, Syrphidae). Dissertação de mestrado, Universidade Federal do Paraná, Curitiba. 2007.

[37] Rech AR, Agostini K, Oliveira PE, Machado IC. Biologia da polinização. Rio de Janeiro: Revisora editorial Ceres Belchior, pp. 527; 2014.

[38] MAPA, Ministério da Agricultura, Pecuária e Abastecimento. Instrução Normativa n° 12, de 28 de março de 2008. D.O.U., Seção 1, de 31 de março de 2008. Available at: http://www.codapar.pr.gov.br/arquivos/File/pdf/FeijaoInstrucaoNormativa1208.pdf

[39] Bragantini C. Alguns aspectos do armazenamento de sementes e grãos de feijão. Embrapa Arroz e Feijão, 187. 2005. Available at: https://www.infoteca.cnptia.embrapa.br/bitstream/doc/194008/1/doc187.pdf. Accessed 11/06/2016.

[40] Burnham KP, Anderson DR. Model selection and multimodel inference: a practical information-theoretic approach. 2rd edn. New York: Springer Verlag; 2002.

[41] Bates D, Maechler M, Bolker B, Walker S. Fitting Linear Mixed-Effects Models using lme4. Journal of Statistical Software. 2015; 67: 1–48. doi: 10.18637/jss.v067.i01

[42] Masiga R, Kasina M, Mbugi J, Odhiambo C, Kinuthia W, Gemmill-Herren B, et al Do French beans (Phaseolus vulgaris) grown in proximity to Mt Kenya forest in Kenya experience pollination deficit? Journal of Pollination Ecology. 2014; 14: 255–260.

[43] Sáez A, Morales CL, Ramos LY, Aizen MA, Steffan‐Dewenter I. Extremely frequent bee visits increase pollen deposition but reduce drupelet set in raspberry. Journal of Applied Ecology. 2014; 51: 1603–1612.

[44] Crane E. Apis species of tropical Asia as pollinators and some rearing methods for them. Acta Hort. 1991; 288: 29–48. doi: 10.17660/ActaHortic.1991.288.2

[45] Roubik DW. Pollination of cultivated plants in the tropics. FAO agricultural services bulletin (299 pp). Rome, Italy; 2005 Available at: http://www.fao.org/3/a-v5040e.pdf

[46] Rusch A, Valantin-Morison M, Sarthou JP, Roger-Estrade J. Effect of crop management and landscape context on insect pest populations and crop damage. Agriculture, Ecosystems and Environment. 2013; 166: 118–125. 10.1016/j.ijcard.2011.10.012

[47] Brittain C, Kremen C, Garber A, Klein AM. Pollination and plant resources change the nutritional quality of almonds for human health. Plos One. 2014; 9: 1–7. 10.1371/journal.pone.0090082 24587215 PMC3937406

[48] Carvalheiro LG, Seymour CL, Nicolson SW, Veldtman R. Creating patches of native flowers facilitates crop pollination in large agricultural fields: mango as a case study. Journal of Applied Ecology. 2012; 49: 1373–1383. 10.1111/j.13652664.2012.02217.x

[49] Brittain C, Williams N, Kremen C, Klein AM. Synergetic effects of non-Apis bees and honey bees for pollination services. Proceedings of The Royal Society B. 2013; 280: 1–8. 10.1098/rspb.2012.2767 PMC3574329 23303545

[50] Hoehn P, Tscharntke T, Tylianakis JM., Steffan-Dewenter I. Functional group diversity of bee pollinators increases crop yield. Proceedings of the Royal Society B. 2008; 275: 2283–2291. 10.1098/rspb.2008.0405 18595841 PMC2603237

[51] Ferreira PA, Boscolo D, Carvalheiro LG, Biesmeijer JC, Rocha PLB, Viana BF. Responses of bees to habitat loss in fragmented landscapes of Brazilian Atlantic Rainforest. Landscape Ecology. 2015; 30: 2067–2078.

[52] Howell AD, Alarcon R, Minckley RL. Effects of habitat fragmentation on the nesting dinamics of desert bees. Annals of the Entomological Society of America. 2017; 110: 233–243.

[53] Simon S, Rusch A, Wyss E, Sarthou J. Conservation Biocontrol: Principles and Implementation in Organic Farming In: Bellon S., Penvern S. Organic farming, prototype for sustainable agriculture (pp. 83–105). Springer, Dordrecht; 2014.

[54] Jauker F, Diekötter T, Schwarzbach F, Wolters V. Pollinator dispersal in an agricultural matrix: opposing responses of wild bees and hoverflies to landscape structure and distance from main habitat. Landscape Ecology. 2009; 24: 547–555. 10.1007/s10980-009-9331-2

[55] Ricketts TH, Regetz J, Dewenter I-S, Cunningham SA, Fremen C, Bogdanski A, et al Landscape effects on crop pollination services: are there general patterns? Ecology Letters. 2008; 11: 499–515. 10.1111/j.1461-0248.2008.01157.x 18294214

[56] Garibaldi LA, Carvalheiro LG, Leonhardt SD, Aizen MA, Blaauw BR, Isaacs R, et al From research to action: enhancing crop yield through wild pollinators. Frontiers in Ecology and Environment. 2014; 12: 439–447. 10.1890/130330

[57] Kremen C, Gonigle LKM. Small-scale restoration in intensive agricultural landscapes supports more specialized and less mobile pollinator species. Journal of applied Ecology. 2015; 55: 602–610. 10.1111/1365-2664.12418

[58] Woodcock BA, Bullock JM, McCracken M, Chapman RE, Ball SL, Edwards ME, et al Spill-over of pest control and pollination services into arable crops. Agriculture, Ecosystems and Environment. 2016; 231: 15–23. 10.1016/j.agee.2016.06.023

[59] Gardener MC, Gillman MP. The effects of soil fertilizer on amino acids in the floral nectar of corncockle, Agrostemma githago (Caryophyllaceae). Oikos. 2001; 92: 101–106. 10.1034/j.1600-0706.2001.920112.x

[60] Gardener MC, Gilman MP. The taste of nectar–a neglected area of pollination ecology. Oikos. 2002; 98: 552–558. http://www.jstor.org/stable/3547198

[61] Nicolson SW. Bee Food: The chemistry and nutritional value of nectar, pollen and mixtures of the two. African Zoology. 2011; 46: 197–204. 10.3377/004.046.0201

[62] Petanidou T, Van Laere A, Ellis WN, Smets E. What shapes amino acid and sugar composition in Mediterranean floral nectars? Oikos. 2006; 115: 155–169. 10.1111/j.2006.0030–1299.14487.x

[63] Bustamante MMC, Medina E, Asner GP, Nardoto GB, Garcia-Montiel DC. Nitrogen cycling in tropical and temperate savannas. Biogeochemistry. 2006; 79: 209–237. 10.1007/s10533-006-9006-x

[64] Soares-Filho B, Rajão R, Macedo M, Carneiro A, Costa W, Coe M, et al Cracking Brazil’s Forest Code. Science. 2014; 344: 363–364. 10.1126/science.1246663 24763575

[65] Zhiyun O, Hua Z, Yi X, Stephen P, Jianguo L, Weihua X, et al Improvements in ecosystem services from investments in natural capital. Science. 2016; 352: 1455–1459. 10.1126/science.aaf2295 27313045

