# Supplementary material for: Crop fertilization affects pollination service provision – Common bean as a case study
Source: PLoS One. 2018 Nov 2;13(11):e0204460. doi: 10.1371/journal.pone.0204460 (PMC6214648; doi:10.1371/journal.pone.0204460)
Supplement: S1 File — All supplementary tables (Tables A to F) and figures (Figures A to C) are listed. Table A. Details of the study areas where flower visitation and yield data were collected. Table B. Management practices applied in each study area that can influence nitrogen (N) and phosphorous (P) content. Table C. Geographical location and soil characteristics of the study areas used in this study. Table D. Identification of the spatial scale at which the different flower visitor groups are most susceptible to changes of vegetation cover. Table E. Details of the species of insects found visiting common bean in our study region. Table F. Sensitivity analyses of the effect of ecosystem services providers (pollinators and biocontrol agents) and nitrogen application on productivity per flower and on overall land productivity. Figure A. Study area located in the central region of Brazil, showing the location of the 35 sampling sites used in this study. Figure B. Effect of the nitrogen input on overall flower production in common bean fields. Figure C. Distribution of pollinator species (native and exotic) and biocontrol agents along a gradient of increasing input of nitrogen (P1 had the highest N input and P35 the lowest). (DOCX) [file pone.0204460.s001.docx]

**S1 File. Combined supporting information file. All supplementary tables (Tables A to G) and figures (Figures A to D) are listed.**

Ramos DL, Bustamante MMC, Silva FDS & Carvalheiro LG. Crop fertilization affects pollination service provision: common bean as a case study

**Table A. Details of the study areas where flower visitation and yield data were collected**. Production deficit was calculated for each sampling site as the ratio between local and maximum production. For each metric and study area, minimum and maximum values obtained per site are presented.

| **Year** | **Study areas** | **Sites** | **Field area (ha)** | **Productivity per flower deficit (%)** (min - max) | **Overall land productivity deficit (%)** (min - max) | **Nitrogen input** (kg/ha) | **Natural vegetation cover** **(%)** (min - max) | **Distance from natural vegetation (meters)** (min - max) |
| --- | --- | --- | --- | --- | --- | --- | --- | --- |
| 2015/  2016 | 1 | 2 | 146.0 | 0 – 8 | 26 – 52 | 106.6* | 0 – 66 | 18 - 565 |
|  | 2 | 2 | 36.0 | 30 – 50 | 22 – 66 | 88.5* | 0 – 24 | 40 - 1045 |
|  | 3 | 3 | 140.0 | 13 – 35 | 0 – 37 | 88.5* | 0 – 11 | 74 - 965 |
|  | 4 | 2 | 39.0 | 50 – 57 | 55 – 58 | 36.0 | 8 – 15 | 57 - 375 |
|  | 5 | 6 | 89.0 | 59 – 85 | 80 – 92 | 98.0* | 3 – 33 | 29 - 375 |
|  | 6 | 2 | 92.0 | 65 – 74 | 84 – 85 | 98.0* | 30 – 53 | 64 - 320 |
|  | 7 | 5 | 145.0 | 22 – 65 | 44 – 76 | 72.6* | 0 – 57 | 97 - 924 |
|  | 8 | 3 | 236.0 | 22 – 47 | 52 – 74 | 91.3* | 0 – 27 | 65 - 939 |
|  | 9 | 2 | 115.0 | 62 – 73 | 65 – 75 | 130.5* | 0 – 50 | 350 - 890 |
| 2016/  2017 | 8 | 4 | 236.0 | 24 – 44 | 47 – 72 | 91.3* | 0 -42 | 46 - 1152 |
|  | 10 | 2 | 35.0 | 23 – 26 | 44 – 49 | 98.7* | 8 – 39 | 100 - 408 |
|  | 11 | 2 | 55.0 | 18 – 27 | 23 – 43 | 98.7* | 5 – 40 | 84 - 371 |

Natural vegetation cover = calculated for each sampling site within a circle of 500 meters radii.

Field area = relates only to the area of the bean field, but other crops were present within the

properties.

‘*’ = refers to study areas with N input above the recommended dosage for common bean plantations in Cerrado (ca. 60 kg ha-1 of nitrogen when area had been previous planted with soya [33]).

**Table B. Management practices applied in each study area that can influence nitrogen (N) and phosphorous (P) content.** For all study areas surrounding vegetation was Cerrado (Brazilian savanna and forest formations), and no tillage was applied in any of the study areas.

| **Study área** | **Year** | **Species used in crop rotation** | **Planting, harvest and spraying dates** | **Fertilization dates** | **Amount of N applied (kg/ha)** | **N source** | **Crop period (N applied)** | **Amount of P applied (kg/ha)** | **P source** | **Crop period (P applied)** |
| --- | --- | --- | --- | --- | --- | --- | --- | --- | --- | --- |
| 1 | 2015 | *Glycine max* (Fabaceae)  *Zea mays* (Poaceae) | **Planting:** 25-oct-2015 **Spraying chemical**: Throughout the planting  **Harvest:** 22-jan-2016 | 25/oct | 106.6 | MAP Ammonium sulfate | MAP (during the planting) Foliar fertilization  10/oct Ammonium sulfate (pre planting) | 46.7 | MAP (P in the form of Phosphate Oxide) | During the planting |
| 2 | 2015 | *Glycine max* (Fabaceae)  *Zea mays*(Poaceae) *Brachiaria ruziziensis* (Poaceae) | **Planting:** 26-oct-2015 **Spraying chemical:** Throughout the planting  **Harvest:** 19-jan-2016 | 05/nov | 88.5 | MAP Nitrate of ammonium | 15/nov (post planting) | 24.23 | MAP (P in the form of Phosphate Oxide) | 15/nov (post planting) |
| 3 | 2015 | *Glycine max* (Fabaceae)  *Zea mays*(Poaceae) *Brachiaria ruziziensis* (Poaceae) | **Planting:** 26-oct-2015 **Spraying chemical:** Throughout the planting  **Harvest:** 19-jan-2016 | 05/nov | 88.5 | MAP Nitrate of ammonium | 15/nov (post planting) | 24.23 | MAP (P in the form of Phosphate Oxide) | 15/nov (post planting) |
| 4 | 2015 | *Fagopyrum tataricum* (Polygonaceae)  *Glycine max* (Fabaceae) *Zea mays* (Poaceae) | **Planting:** 1-oct-2015 **Spraying chemical:** Throughout the planting  **Harvest:** 29-jan-2016 | 23/sep: KCl 1/oct: NPK | 36 | MAP | During the planting | 65,3 | MAP and reactive fosfate; P in the form of Phosphate Oxide | MAP (during the planting); reactive fosfate (pre planting) |
| 5 | 2015 | *Zea mays* (Poaceae) *Crotelaria* sp. (Fabaceae) Millet (Poaceae) | **Planting:** 1-nov-2015 **Spraying chemical:** Throughout the planting  **Harvest:** 30-jan-2016 | 01/nov | 98 | MAP  Urea | Urea (post planting) NPK (during the planting) | 32.31 | MAP P in the form of Phosphate Oxide | During the planting |
| 6 | 2015 | *Zea mays* (Poaceae) *Crotelaria* sp. (Fabaceae) Millet (Poaceae) | **Planting:** 1-nov-2015 **Spraying chemical:** Throughout the planting  **Harvest:** 30-jan-2016 | 01/nov | 98 | MAP  Urea | Urea (post planting) NPK (during the planting) | 32.31 | MAP P in the form of Phosphate Oxide | During the planting |
| 7 | 2015 | *Zea mays* (Poaceae) *Glycine max* (Fabaceae) | **Planting:** 06-nov-2015 **Spraying:** chemical: Throughout the planting  **Harvest:** 22-feb-2016 | 02/nov - Nitrate 04/nov –NPK KCl (pre planting) | 72.6 | MAP Nitrate of ammonium 27% | Nitrate of ammonium (pre planting); pre planting) and foliar (post-planting) | 30.38 | MAP P in the form of Phosphate Oxide | Pre-planting – MAP and foliar (post planting) |
| 8 | 2015 | *Glycine max* (Fabaceae) *Zea mays* (Poaceae)  *Crotelaria* sp. (Fabaceae)  Millet (Poaceae)  *Brachiaria ruziziensis* (Poaceae) | **Planting:** 1-nov-2015 **Spraying:** chemical: Throughout the planting  Harvest: 28-jan-2016 | 1/nov -  Fertilizers KCl (pre-planting) | 91.3 | Urea | During the planting | 41.48 | MAP P in the form of Phosphate Oxide | During the planting |
| 10 | 2015 | *Fagopyrum tataricum* (Polygonaceae) *Glycine max* (Fabaceae) *Zea mays* (Poaceae)  *Avena strigosa* (Poaceae) | **Planting:** 27-nov-2015 **Spraying chemical:** Throughout the planting  **Harvest:** 22-feb-2016 | 27/nov:  NPK Urea (20 days after the emergence) | 130.5 | MAP  Urea | During the planting and pre planting (manure) | 40.39 | MAP P in the form of Phosphate Oxide | During the planting |
| 8 | 2016 | *Zea mays* (Poaceae) *Triticum* sp. (Poaceae) *Glycine max* (Fabaceae) | **Planting:** 30-oct-2016 **Spraying chemical:** Throughout the planting  **Harvest:** 20-jan-2017 | NPK (during the planting) KCl (pre planting) | 91.3 | Urea | During the planting | 41.48 | MAP P in the form of Phosphate Oxide | During the planting |
| 10 | 2016 | *Triticum sp*. (Poaceae) *Sorghum bicolor* (Poaceae) | **Planting:** 26-oct-2016 **Spraying chemical:** Throughout the planting  **Harvest:** 20-jan-2017 | 20/oct: KCl 27/10: NPK | 98.7 | MAP Nitrate of ammonium 27% | NPK: (during the planting) Nitrate and ammonium sulfate (post planting) | 36.4 | MAP P in the form of Phosphate Oxide | 27/10 -MAP; P51(Foliar): post planting |
| 11 | 2016 | *Triticum sp*. (Poaceae) *Sorghum bicolor* (Poaceae) | **Planting:** 26-oct-2016 **Spraying chemical:** Throughout the planting;  **Harvest:** 20-jan-2017 | 20/oct: KCl 27/10: NPK | 98.7 | MAP Nitrate of ammonium 27% | NPK: (during the planting) Nitrate and ammonium (post planting) | 36.4 | MAP P in the form of Phosphate Oxide | 27/10 - MAP; P51(Foliar): post planting |

**Table C. Geographical location and soil characteristics of the study areas used in this study.** Soil from all study areas is classified as Latossolo vermelho (Brazilian Soil Taxonomy) and Oxisols (US Soil Taxonomy). Due to logistic problems soil samples were not collected in site 1. Values represent minimum and maximum values obtained, except for study areas where all samples had the same value (*).

| **Year** | **Study area** | **Coordinates** | **Soil characteristcs (0-20 cm depth)** | | | | |
| --- | --- | --- | --- | --- | --- | --- | --- |
|  |  |  | pH (H_2_O) (min - max) | pH (CaCl_2_) (min - max) | Cation exchange capacity - ECEC (min-max) (cmolc/dm³) | Organic matter (min-max) (dag/kg) | Base saturation (min-max) (%) |
| 2015/2016 | 1 | 16°07'16.8"S 47°52'57.2"W | NA | NA | NA | NA | NA |
|  | 2 | 15°55'06.2"S 47°25'53.6"W | 5.2-5.3 | 4.6-5.1 | 5.0-6.0 | 4.9* | 49.5-55.8 |
|  | 3 | 16°13'00.1"S 47°32'45.6"W | 5.2-6.1 | 5.1-6.2 | 6.0 - 7.1 | 3.9-5.1 | 63.3-87.5 |
|  | 4 | 15°51'49.3"S 47°36'34.5"W | 4.9-5.8 | 4.9-5.5 | 6.2-6.7 | 4.8-6.0 | 59.6-64.1 |
|  | 5 | 15°58'37.2"S 47°34'37.9"W | 4.7-5.1 | 4.4-4.9 | 6.1-9.0 | 3.8-4.8 | 63.0-74.4 |
|  | 6 | 15°58'37.2"S 47°34'37.9"W | 4.7-5.1 | 4.4-4.9 | 4.8-6.8 | 4.6-4.9 | 49.1-70.8 |
|  | 7 | 15°45'54.8"S 47°19'57.8"W | 4.7-5.3 | 4.9-5.3 | 3.6-4.9 | 4.1-4.8 | 35.9-52.2 |
|  | 8 | 15°52'04.0"S 47°33'39.2"W | 5.0-5.7 | 5.1-5.8 | 6.5-8.3 | 4.3-5.3 | 62.0-73.0 |
|  | 9 | 15°41'50.0"S 47°30'13.5"W | 4.9 | 4.5-4.6 | 4.1-5.2 | 2.9-4.0 | 45.5-53.2 |
| 2016/2017 | 8 | 15°52'04.0"S 47°33'39.2"W | 4.5-5.1 | 4.3-4.6 | 5.2-9.1 | 4.7-5.2 | 49.6-60.0 |
|  | 10 | 15°51'40.0"S 47°35'27.4"W | 4.9-5.1 | 4.8-4.9 | 5.5-5.7 | 3.6-4.7 | 39.7-43.1 |
|  | 11 | 15°50'40.8"S 47°34'40.5"W | 4.7-4.8 | 4.6* | 7.5-7.8 | 3.7-4.8 | 61* |

**Table D. Identification of the spatial scale at which the different flower visitor groups are most susceptible to changes of vegetation cover.** Values of the Akaike Information Criterion corrected for small sample size are presented for each model. Spatial scale selected for each group is marked in bold and was used in subsequent data analyses.

| **Landscape variable in different spatial scales** | **Native pol.** | **Exotic pol.** | **Bio.control** | | **Pest** | **Richness** |
| --- | --- | --- | --- | --- | --- | --- |
| Vegetation cover (0.5 km) | 148.81 | **159.43** | | **140.01** | **73.44** | **152.20** |
| Vegetation cover (1 km) | 146.06 | 159.90 | | 144.72 | 74.20 | 156.79 |
| Vegetation cover (1.5 km) | 144.28 | 161.22 | | 145.74 | 73.78 | 158.48 |
| Vegetation cover (2.0 km) | **141.48** | 161.15 | | 145.04 | 73.47 | 155.94 |

**Table E. Details of the species of insects found visiting common bean in our study region.** Overall 283 visitors were detected. The main ecological function of each species was defined based on the behaviour described in the indicated references.

| **Flower visitor (species or morphospecies)** | **Origin** | **Observed behaviour** | **Overall frequency of visits (%)** | **Main ecological function** | **Reference** |
| --- | --- | --- | --- | --- | --- |
| HYMENOPTERA |  |  |  |  |  |
| Apidae |  |  |  |  |  |
| *Paratrigona lineata*  Lepeletier, 1836 | Native | Legitimate Floral Visitor | 14.8 | Pollinator | [1] |
| *Apis melífera*  Linnaeus, 1758 | Exotic | Legitimate Floral Visitor / Robber | 32.5 | Pollinator | [1] |
| *Bombus (Fervidobombus) morio*  Swederus, 1787 | Native | Legitimate Floral Visitor | <1 | Pollinator | [1] |
| *Trigona spinipes*  Fabricius, 1793 | Native | Legitimate Floral Visitor /Robber | 3.5 | Pollinator | [1;2] |
| *Bombus brevivillus cf.*  Franklin, 1913 | Native | Legitimate Floral Visitor | 1.4 | Pollinator | [3] |
| *Bombus sp.* | Native | Legitimate Floral Visitor | 1.4 | Pollinator | [3] |
| *Geotrigona sp.* | Native | Legitimate Floral Visitor | <1 | Pollinator | [2] |
| *Exomalopsis analis*  Spinola, 1853 | Native | Legitimate Floral Visitor | <1 | Pollinator | [1] |
| *Xylocopa (Stenoxylocopa) sp.* | Native | Legitimate Floral Visitor | <1 | Pollinator | [4] |
| *Apoidea sp.* | No information | Legitimate Floral Visitor | 8.5 | Pollinator |  |
| Andrenidae (Oxaeinae) |  |  |  |  |  |
| *Oxaea flavescens*  Klug, 1807 | Native | Legitimate Floral Visitor | <1 | Pollinator | [5] |
| Vespidae |  |  |  |  |  |
| *Polybia ignobilis*  Haliday, 1836 | Native | Robber | <1 | Predator | [6] |
| *Brachygastra lecheguana*  Latrille, 1824 | Native | Legitimate Floral Visitor /Robber | 1.4 | Predator | [7] |
| *Polybia dimidiata*  Oliver, 1791 | Native | Robber | <1 | Predator | [8] |
| Pompilidae |  |  |  |  |  |
| *Pepsis* sp. | No information | Robber | < 1 | Predator | [9] |
|  |  |  |  |  |  |
| DIPTERA |  |  |  |  |  |
| Syrphidae |  |  |  |  |  |
| *Allograpta cf. exotica*  Wiedemann, 1830 | Exotic | Legitimate Floral Visitor | 28.3 | Predator | [10] |
| *Palpada vinetorum*  Fabricius, 1798 | Exotic | Legitimate Floral Visitor | <1 | Pollinator | [11] |
| Ulidiidae |  |  |  |  |  |
| *Euxesta sp.* | No information | Legitimate Floral Visitor | <1 | - |  |
| Drosophilidae |  |  |  |  |  |
| Drosophilidae sp. | No information | Legitimate Floral Visitor | <1 | - |  |
|  |  |  |  |  |  |
| LEPIDOPTERA |  |  |  |  |  |
| Noctuidae |  |  |  |  |  |
| *Heliothis virescens*  Fabricius, 1777 | Exotic | Legitimate Floral Visitor | <1 | Pest (tobacco, bean, soybean) | [12; 13] |
| *Helicoverpa zea*  Boddie, 1850 | Exotic | Legitimate Floral Visitor | 1.4 | Pest (tomato, corn, bean) | [8;13] |
| Hesperiidae |  |  |  |  |  |
| *Urbanus proteus*  Linnaeus, 1758 | Exotic | Legitimate Floral Visitor | <1 | Pest (bean) | [13;14] |
| *Urbanus d. dorantes*  Stoll, 1790 | Exotic | Legitimate Floral Visitor | <1 | Pest (bean, soybean) | [13] |
| *Hylephila p. phyleus*  Drury, 1773 | Exotic | Legitimate Floral Visitor | <1 | - |  |
| *Panoquina lucas*  Fabricius, 1793 | Exotic | Legitimate Floral Visitor | <1 | - |  |
| *Aguna asande*  Hewitson, 1867 | Exotic | Legitimate Floral Visitor | <1 | - |  |
| *Chioides c. catillus*  Cramer, 1780 | Exotic | Legitimate Floral Visitor | <1 | Pest (soybean) | [13] |
| Pieridae |  |  |  |  |  |
| *Eurema e. elathea*  Cramer, 1777 | Exotic | Legitimate Floral Visitor | <1 | Pest (bean, soybean) | [13] |
| Arctiidae |  |  |  |  |  |
| Arctiidae (morpho1) | No information | Legitimate Floral Visitor | <1 | - |  |
|  |  |  |  |  |  |
| COLEOPTERA |  |  |  |  |  |
| Chrysomelidae |  |  |  |  |  |
| *Diabrotica speciosa*  German, 1824 | Native | Legitimate Floral Visitor / Herbivorous | <1 | Pest(bean, soybean, tomato, corn) | [14; 15] |
| *Diabrotica sp.1* | No information | Legitimate Floral Visitor | <1 | Pest | [14] |
| Curculionidae |  |  |  |  |  |
| - Curculionidae sp. | No information | Legitimate Floral Visitor | <1 | - | - |
| HEMIPTERA |  |  |  |  |  |
| Pentatomidae |  |  |  |  |  |
| - *Euschistus heros*  Fabricius, 1794 | Exotic | Robber | <1 | Pest (bean, soybean, cotton) | [14] |
| - Hemiptera (morpho 1) | No information | Robber | <1 | - |  |
| - Hemiptera (morpho 2) | No information | Robber | <1 | - |  |

*Supplementary references:

[1] Santana MP, Carvalho CF, Souza B, Morgado LN. Abelhas (Hymenoptera: Apoidea) visitantes de flores do feijoeiro, *Phaseolus vulgaris* L., em Lavras e Ijaci – MG. Ciência e Agrotecnologia. 2002; 26: 1119-1127.

[2] Heard TA. The role of stingless bees in crop pollination. Annual Review of Entomology. 1999; 44: 183-206.

[3] Hoc PS, & García MTA. Biología floral y sistema reproductivo de *Phaseolus vulgaris* var. Aborigineus (Fabaceae). Revista de Biologia Tropical. 1999; 47: 59-67.

[4] Kingha BMT, Fohouo F-NT, Ngakou A, Brückner D. (2012). Foraging and pollination activities of *Xylocopa olivacea* (Hymenoptera, Apidae) on *Phaseolus vulgaris* (Fabaceae) flowers at Dang (Ngaoundere- Cameroon). Journal of Agricultural Extension and Rural Development. 2012; 4: 330-339.

[5] Forni-Martins ER, Marques MCM, Lemes MR. Biologia floral e reprodução de *Solanum paniculatum* L. (Solanaceae) no estado de São Paulo, Brasil. Brazilian Journal of Botany. 1998; 21: 117-124.

[6] Picanço M, Oliveira IR, Rosado JF, Silva RS. Natural Biological Control of Ascia monuste by the Social Wasp *Polybia ignobilis* (Hymenoptera: Vespidae). Sociobiology. 2010; 56: 67-76.

[7] Fernandes FL, Da Silva PR, Gorri J, Pucci LF, Da Silva IW. Selectivity of old and new organophosphate insecticides and behavior of Vespidae predators in coffee crop. Sociobiology. 2013; 60: 471-476.

[8] Campos-Farinha AEC, Pinto NPO. Natural enemies of *Chlosyne lacinia saundersii* Doubl. & Hew. (Lepidoptera: Nymphalidae) in the State of São Paulo. Anais da Sociedade Entomológica do Brasil. 1996; 25: 165-168.

[9] Corseuil E. Controle biológico. Entomologia – Temas didáticos N° 1. Porto Alegre. 2007.

[10] Oliveira MRV, Amancio E, Laumann RA, Gomes L de O. Natural enemies of *Bemisia tabaci* (Gennadius) *B Biotype* and *Trialeurodes vaporariorum* (Westwood) (Hemiptera: Aleyrodidae) in Brasília, Brazil. Neotropical Entomology. 2003; 32: 151 – 154.

[11] Costa JNM, Brum CM, Júnior JRV, Faria GV, Uchoa TL, Silva TT. Ocorrência de polinizador de café *Palpada vinetorum* (DIPTERA: Shyrphidae) e parasitismo pelo fungo Cordyceps sp. IX Simpósio de Pesquisa dos Cafés do Brasil. Curitiba. 2015.

[12] Bortolotto OC, Bueno AF, Braga K, Biato R, Queiroz AP, Pomari AF, et al. Parâmetros biológicos de *Heliothis virescens* (Lepidoptera: Noctuidae) em folha e vagem de feijão. XXV Congresso Brasileiro de Entomologia. 2014.

[13] Formentini AC. Lepidópteros associados à cultura da soja: diversidade e parasitismo natural por insetos e fungos entomopatogênicos. M.Sc. Thesis, University of Caxias do Sul, Caxias do Sul. 2009.

[14] Quintela ED. Manual de identificação de insetos e outros invertebrados pragas do feijoeiro. Documentos - Embrapa Arroz e Feijão, ISSN 1678-9644, Santo Antônio de Goiás, GO. p. 68; 2009.

[15] Cividanes FJ, & Yamamoto FT. Pragas e inimigos naturais na soja e no milho cultivados em sistemas diversificados. Scientia Agricola. 2002; 59: 683-687.

**Table F. Sensitivity analyses of the effect of ecosystem services providers (pollinators and biocontrol agents) and nitrogen application on productivity per flower and on overall land productivity.** Analyses were repeated first without the two sites with lowest N input (36 kg ha^-1^), and then without the site with highest N input (130.5 kg ha^-1^). Akaike information criterion corrected for small sample size (AICc), variation of AICc to the best model (∆AICc), and AICc weights are provided for all models with ∆AICc lower than 2.2 (due to the reduced number of points, we opted for a more inclusive approach, using 2.2 instead of 2 as cut off value).

| **Response variable (Y)** | **Explanatory variables** | | | | | **Weight** | **AICc** | **∆AICc** |
| --- | --- | --- | --- | --- | --- | --- | --- | --- |
| PRODUCTIVITY PER FLOWER | DN | DE | N | DN*N | BCT |  |  |  |
| Without low N sampling sites |  | | | | | | | |
| Model 1 | - | - | - | - | - | 0.291 | 59.7 | 0.00 |
| Model 2 | - | X | - | - | - | 0.138 | 61.3 | 1.51 |
| Average model | log(Y/(2-Y))=-0.08 -42.80*DE | | | | | | | |
| Without high N sampling sites | | | | | | | | |
| Model1 | - | - | X | - | - | 0.364 | 57.4 | 0.00 |
| Model2 | X | - | X | X | - | 0.126 | 59.6 | 2.11 |
| Average model | log(Y/(2-Y))=-=-1.6+(650.0-6.9*N)*DN+0.017*N | | | | | | | |
| OVERALL LAND PRODUCTIVITY |  | | | | | | | |
| Without low N sampling sites |  | | | | | | | |
| Model 1 | - | - | - | - | - | 0.313 | 106.4 | 0.00 |
| Model 2 | - | X | - | - | - | 0.150 | 107.9 | 1.47 |
| Model 3 | X | - | - | - | - | 0.123 | 108.3 | 1.87 |
| Average model | log(Y/(5300-Y))= -1.11 -97.57*DE+48.29*DN | | | | | | | |
| Without high N sampling sites |  | | | | | | | |
| Model 1 | - | - | - | - | - | 0.312 | 105.7 | 0.00 |
| Model 2 | X | - | - | - | - | 0.135 | 107.4 | 1.68 |
| Model 3 | - | - | X | - | - | 0.106 | 107.8 | 2.17 |
| Average model | log(Y/(5300-Y))= -0.25-52.93*DN+0.01*N | | | | | | | |

Low N points = sampling sites with nitrogen application until 36 kg/ha

High N points = N application was of 130.5 kg/ha

RF = richness

DN = density of native pollinators

DE = density of exotic pollinators (*A. mellifera*)

BCT = density of biocontrol agents

N = input of nitrogen

‘*’ = represents the two-way interaction between explanatory variables

‘x’= indicates terms that were included in the models (Gaussian distribution for productivity per flower, and negative binomial for overall land productivity).

**Table G – Effect if landscape variables, nitrogen input on abundance of ecosystem providers, taking into account the effect of climatic variables (temperature, humidity and wind speed), observation time and number of flowers**.

| **Response (Y)** | **Terms** | | | | | | | | |  | | **Weight** | **AICc** | | **∆AICc** |  |
| --- | --- | --- | --- | --- | --- | --- | --- | --- | --- | --- | --- | --- | --- | --- | --- | --- |
| **Nat pol** | VC | D | N | VC*N | NFL | HU | TEMP | OT | WS | |  | |  |  | |  |
| Model 1 | X | X | - | - | - | X | - | - | - | | 0.233 | | 180.3 | 0.00 | |  |
| Model 2 | - | X | - | - | - | X | - | - | - | | 0.167 | | 181.0 | 0.66 | |  |
| Model 3 | X | X | - | - | - | X | X | - | - | | 0.098 | | 182.0 | 1.74 | |  |
| Average model Y=e^(-1.04-0.004*D-0.0567*VC+0.0419*HU+0.0911*TEMP)^ | | | | | | | | | | | | | | | | |
| **Exotic pol** | VC | D | N | VC*N | NFL | HU | TEMP | OT | WS | |  | |  |  | |  |
| Model 1 | - | - | X | - | X | X | - | - | - | | 0.181 | | 213.4 | 0.00 | |  |
| Model 2 | X | - | X | X | X | - | - | - | - | | 0.145 | | 213.8 | 0.44 | |  |
| Model 3 | - | - | X | - | X | - | - | - | - | | 0.139 | | 213.9 | 0.52 | |  |
| Model 4 | X | - | X | X | X | X | - | - | - | | 0.129 | | 214.0 | 0.67 | |  |
| Model 5 | - | - | X | - | X | X | - | X | - | | 0.083 | | 214.9 | 1.55 | |  |
| Model 6 | X | - | X | - | X | - | - | - | - | | 0.081 | | 215.0 | 1.60 | |  |
| Model 7 | X | - | X | - | X | X | - | - | - | | 0.069 | | 215.3 | 1.91 | |  |
| Average model Y=e^(-3.51+0.0176*N+0.0005*NFL+0.0240*HU-(0.0501+0.0009*N)*VC+0.00007*OT)^ | | | | | | | | | |  | |  |  | | |  |
| **BC Abund** | VC | D | N | VC*N | NFL | HU | TEMP | OT | WS | |  | |  |  | |  |
| Model 1 | - | - | X | - | - | - | - | - | - | | 0.098 | | 199.8 | 0.00 | |  |
| Model 2 | X | - | X | - | - | X | - | X | - | | 0.081 | | 200.2 | 0.38 | |  |
| Model 3 | - | - | X | - | - | - | - | X | - | | 0.076 | | 200.3 | 0.49 | |  |
| Model 4 | X | - | X | - | - | - | - | - | - | | 0.067 | | 200.6 | 0.75 | |  |
| Model 5 | X | X | X | - | - | X | - | X | - | | 0.055 | | 201.0 | 1.15 | |  |
| Model 6 | - | - | X | - | - | - | - | - | X | | 0.052 | | 201.1 | 1.25 | |  |
| Model 7 | X | - | X | - | - | - | - | X | - | | 0.051 | | 201.2 | 1.28 | |  |
| Model 8 | X | X | X | - | - | - | - | X | - | | 0.049 | | 201.3 | 1.37 | |  |
| Model 9 | - | - | - | - | X | - | - | - | - | | 0.046 | | 201.4 | 1.49 | |  |
| Model 10 | X | X | X | - | - | - | - | - | - | | 0.046 | | 201.5 | 1.51 | |  |
| Model 11 | - | - | X | - | X | - | - | - | - | | 0.042 | | 201.6 | 1.69 | |  |
| Model 12 | X | - | X | X | - | - | - | - | - | | 0.041 | | 201.6 | 1.73 | |  |
| Model 13 | - | - | - | - | - | - | - | - | - | | 0.040 | | 201.6 | 1.77 | |  |
| Model 14 | X | X | X | - | - | - | X | X | - | | 0.039 | | 201.7 | 1.83 | |  |
| Model 15 | X | - | X | X | - | X | - | X | - | | 0.039 | | 201.7 | 1.85 | |  |
| Model 16 | X | - | X | - | - | - | - | - | X | | 0.038 | | 201.7 | 1.90 | |  |
| Average model Y=e^(3.86-0.0383*N+0.00006*NFL-0.0021*OT-0.0494*HU+(0.0157+0.00006*N)*VC+0.0013*D-0.2799*WS+0.1332*TEMP)^ | | | | | | | | | | | | | | | | |
| **Pest** | VC | D | N | VC*N | NFL | HU | TEMP | OT | WS | |  | |  |  | |  |
| Model 1 | - | - | - | - | - | - | - | - | - | | 0.153 | | 73.7 | 0.00 | |  |
| Model 2 | - | - | - | - | - | - | - | X | - | | 0.094 | | 74.6 | 0.97 | |  |
| Model 3 | - | - | - | - | - | - | - | - | - | | 0.075 | | 75.1 | 1.42 | |  |
| Model 4 | - | - | - | - | - | - | - | X | - | | 0.063 | | 75.4 | 1.78 | |  |
| Model 5 | - | - | - | - | X | - | - | - | - | | 0.061 | | 75.5 | 1.85 | |  |
| Model 6 | - | - | X | - | - | - | - | - | - | | 0.060 | | 75.5 | 1.88 | |  |
| Average model logit(Y)=0.13+2.5856*NFL-0.0136*N-0.0021*OT-0.7160*WS | | | | | | | | | | | | | | | | |
| **Richness** | VC | D | N | VC*N | NFL | HU | TEMP | OT | WS | |  | |  |  | |  |
| Model 1 | X | - | - | - | - | - | - | - | X | | 0.107 | | 240.9 | 0.00 | |  |
| Model 2 | X | - | X | - | - | - | - | - | X | | 0.079 | | 241.5 | 0.60 | |  |
| Model 3 | X | - | X | - | - | - | - | - | - | | 0.074 | | 241.6 | 0.72 | |  |
| Model 4 | X | - | - | - | - | - | - | X | X | | 0.068 | | 241.8 | 0.90 | |  |
| Model 5 | X | - | - | - | - | - | - | - | - | | 0.067 | | 241.8 | 0.92 | |  |
| Model 6 | X | - | X | - | - | - | - | X | - | | 0.062 | | 242.0 | 1.08 | |  |
| Model 7 | X | - | - | - | X | - | - | - | X | | 0.058 | | 242.1 | 1.21 | |  |
| Model 8 | X | - | - | - | - | - | - | X | - | | 0.055 | | 242.2 | 1.33 | |  |
| Model 9 | X | - | X | - | - | - | - | X | X | | 0.054 | | 242.3 | 1.34 | |  |
| Model 10 | X | X | - | - | - | - | - | - | X | | 0.053 | | 242.3 | 1.42 | |  |
| Model 11 | X | - | X | - | - | X | - | - | X | | 0.044 | | 242.7 | 1.79 | |  |
| Model 12 | X | - | X | - | X | - | - | - | X | | 0.041 | | 242.8 | 1.92 | |  |
| Average model Y=e^(1.13+0.0152*VC-0.0070*N+1.0593*NFL-0.1525*WS-0.0005*OT-0.00003*D+0.0091*HU)^ | | | | | | | | | | | | | | | | |

All models were run assuming negative binomial distribution, except for pests for which a binomial model was applied. The spatial scale used in vegetation cover (VC) was 500 meters for models of biocontrol agents, exotic pollinator, richness and pests (probability of occurrence) while for native pollinators was 2000 meters (see the *a priori* VC model selection procedure in Table D).

* = represents the two-way interaction between explanatory variables; X = indicates terms that were included in the models; N = Nitrogen input; D = Distance from native vegetation; VC = Native vegetation cover; NFL = Number of flowers observed during surveys; TEMP = Temperature; H = Humidity; WS = Wind speed; OT = Observation time; NFL = Number of flowers observed during surveys


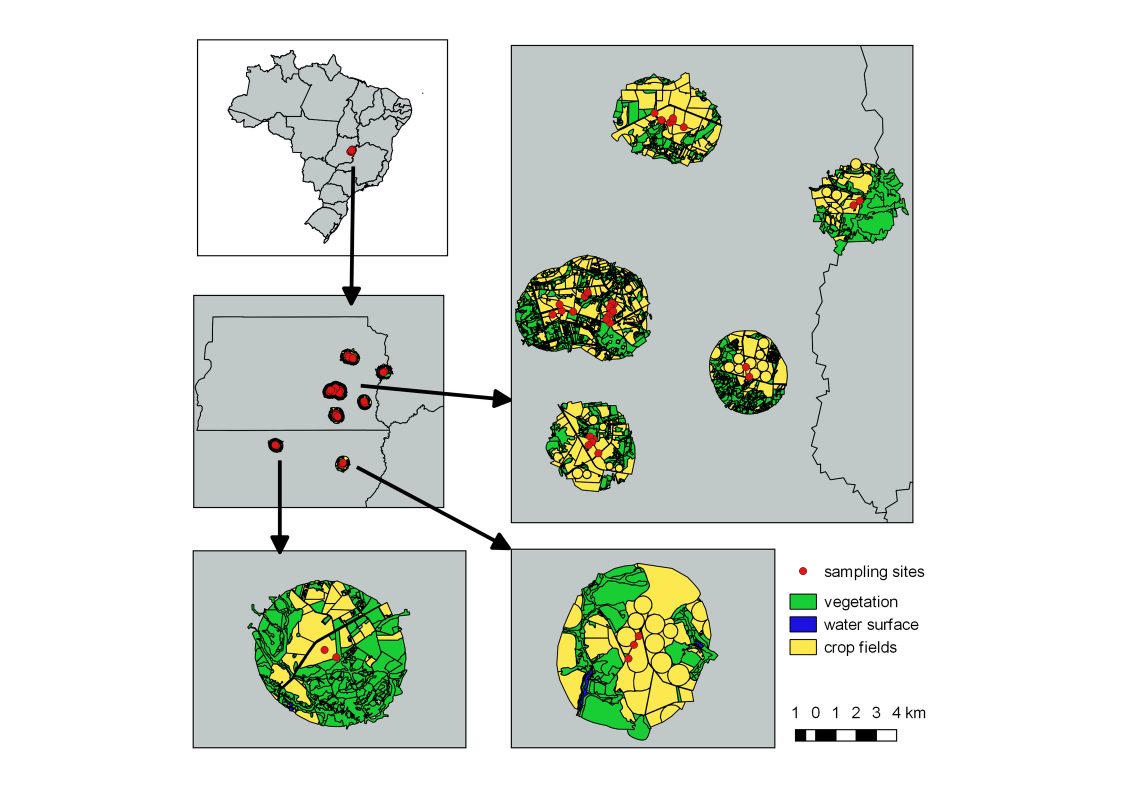


**Figure A. Study area located in the central region of Brazil, showing the location of the 35 sampling sites used in this study**. This area is characterized by high degree of land conversion, with large monocultures. The image provides an example of buffers (3500 meters radii) with land-use classes selected around the fields.


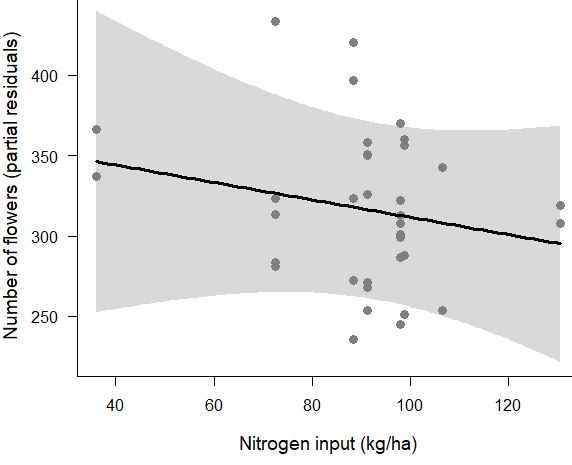


**Figure B. Effect of the nitrogen input on overall flower production (per m2) in common bean fields.** The effect is negative and the P-value from log-likelihood ratio test between linear mixed models with and without the variable in the model was 0.42. Year and farm within farmer were included in the models as random effects.

**
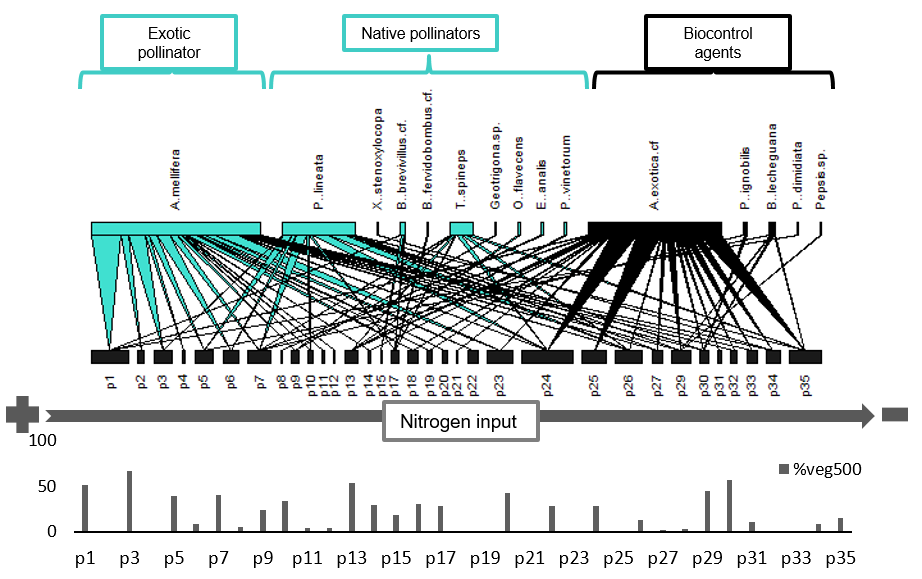
**

**Figure C. Distribution of pollinator species (native and exotic) and biocontrol agents along a gradient of increasing input of nitrogen (P1 had the highest N input and P35 the lowest).** The width of the boxes in the top level (flower visitors) is proportional to the overall abundance of the species. The width of the boxes in the lower level is proportional to the visitation rates within each sampling site. The graph on the bottom shows the native vegetation cover (%) within 500m of each sampling site.


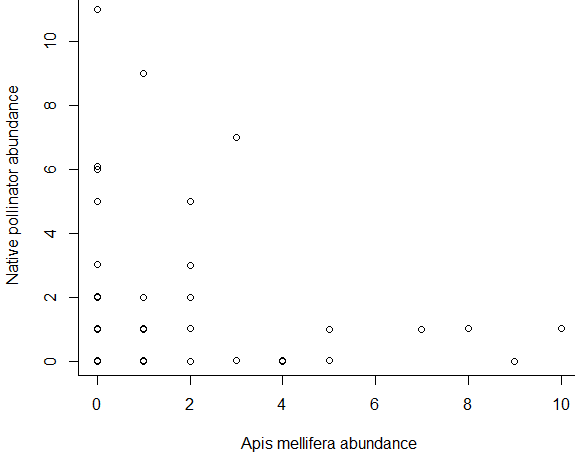


**Figure D. Effect of *Apis mellifera* density on native pollinator density.** The effect was tested using generalized linear mixed models, with negative binomial family, and year and farm within farmer as random effects. The estimate of the effect was -0.12±0.10 (standard error), and a loglikelihood test with and without the variable was non-significant (p-value=0.223).
